# Unlocking the Antioxidant Potential of Sea Cucumber Viscera: Pre-Treatment Modulates the Keap1-Nrf2 Pathway and Gut Microbiota to Attenuate Cold Stress-Induced Oxidative Damage

**DOI:** 10.3390/antiox14111355

**Published:** 2025-11-13

**Authors:** Yang Gao, Xin Qiao, Xueyi Jing, Weiyue Li, Dongchao Zhang, Lei Pu, Jianbin Zhang, Hua Yang, Xingyao Pei, Liang Hong

**Affiliations:** Tianjin Key Laboratory of Agricultural Animal Breeding and Healthy Husbandry, College of Animal Science and Veterinary Medicine, Tianjin Agricultural University, Tianjin 300392, China; gy13512866631@163.com (Y.G.); tjau_qiaoxin@163.com (X.Q.); jingxy315@163.com (X.J.); liweiyue925@163.com (W.L.); zdc@tjau.edu.cn (D.Z.); puleiwork@tjau.edu.cn (L.P.); zjb@tjau.edu.cn (J.Z.); yanghua@tjau.edu.cn (H.Y.)

**Keywords:** sea cucumber viscera, oxidative stress, Keap1-Nrf2/HO-1 pathway, gut microbiota

## Abstract

The internal organs of sea cucumbers (SCV) are a byproduct of the seafood processing industry and hold untapped potential as a functional food. This study investigates the antioxidant capacity of SCV and its regulatory effects on the gut microbiota in a mouse model of oxidative stress induced by chronic cold exposure. The results indicate that SCV possesses a rich nutritional composition, containing various components such as calcium, phosphorus, and polysaccharides, and exhibit strong scavenging activity against three types of free radicals in vitro: DPPH, OH^−^, and O_2_^−^. SCV significantly reduced MDA levels in both serum and liver, while activating the Keap1-Nrf2/HO-1 pathway, leading to a significant decrease in the expression of HSP70 and HSP90 genes and a marked increase in Nrf2 gene expression, thereby alleviating oxidative damage. Histological analysis revealed that SCV alleviated liver damage, reducing hepatocellular vacuolization and inflammatory cell infiltration. Additionally, SCV modulated the diversity of the gut microbiota, increasing the abundance of *Allobaculum*, *Turicibacter*, *Bifidobacterium*, and *Akkermansia*, while enriching the synthesis pathway of vitamin B12 (PWY-7377). This study is the first to repurpose sea cucumber viscera waste into a functional food, demonstrating its dual mechanism of alleviating oxidative stress by activating the Keap1-Nrf2/HO-1 antioxidant pathway and regulating the gut microbiota. These findings offer an innovative strategy for the high-value utilization of agricultural by-products and the development of multifunctional health-promoting products.

## 1. Introduction

Oxidative stress arises when the generation of oxidants such as reactive oxygen species (ROS) and reactive nitrogen species (RNS) exceeds the scavenging capacity of endogenous antioxidant defense systems, leading to damage of cellular macromolecules and contributing to the progression of various diseases [1,2,3]. In homeothermic animals, chronic cold stress represents a typical environmental challenge that induces systemic oxidative stress [4]. To maintain a stable core body temperature, the organism initiates a series of adaptive thermogenic responses. Tissues such as brown/beige adipose tissue, liver, and skeletal muscle enhance mitochondrial heat production through the activation of uncoupling proteins (UCPs) located on the inner mitochondrial membrane [5,6,7]. UCP1 uncouples oxidative phosphorylation by dissipating the proton gradient across the mitochondrial membrane, thereby converting energy from substrate oxidation directly into heat [8,9,10,11]. This process is accompanied by a dramatic increase in metabolic rate, necessitating the mobilization and oxidation of substantial fat stores to provide sustained fuel [12,13,14]. In addition to UCP1, other UCP family members, such as UCP3, also contribute significantly to energy metabolism and thermoregulation [15,16].

However, the elevated metabolic activity and enhanced mitochondrial electron transport also lead to increased electron leakage, resulting in overproduction of ROS [17,18,19,20]. While ROS at physiological levels act as important signaling molecules regulating processes such as growth and proliferation, excessive ROS disrupt cellular redox homeostasis and trigger oxidative stress [21]. Mitochondria are not only the primary source of ROS but also a major target of oxidative damage [22]. Under conditions of oxidative stress, mitochondrial components—including mtDNA, functional proteins, and membrane lipids—can be impaired, which in turn exacerbates mitochondrial dysfunction and promotes further ROS generation, creating a vicious cycle of oxidative damage [23]. Thus, under cold stress, enhancing the antioxidant defense system is essential to mitigate the risks of oxidative damage.

The Keap1-Nrf2/HO-1 signaling axis serves as a central regulatory pathway in the cellular defense against oxidative stress [24]. Under basal conditions, the transcription factor Nrf2 is constitutively bound by its cytosolic repressor Keap1, which promotes its ubiquitination and proteasomal degradation, thereby maintaining low cellular levels of Nrf2 [25]. Upon oxidative challenge, elevated ROS levels promote the phosphorylation and subsequent dissociation of Nrf2 from Keap1. The liberated Nrf2 then translocates into the nucleus, where it binds to antioxidant response elements (AREs) in the promoter regions of various cytoprotective genes, activating their transcription [26]. HO-1 represents a key downstream effector. The catalytic products of HO-1, biliverdin and carbon monoxide (CO), exhibit potent antioxidant and anti-inflammatory properties, constituting an essential mechanism to counteract oxidative injury [27]. Notably, Nrf2 activation also upregulates the expression and function of uncoupling protein 3 (UCP3). Emerging evidence indicates that UCP3 is a significant transcriptional target of Nrf2, and its induction helps mitigate mitochondrial oxidative stress [28]. This implies that Nrf2 pathway activation not only enhances classical antioxidant responses via HO-1 but may also attenuate mitochondrial ROS production at the source through UCP3 upregulation, collectively forming an integrated, multi-layered defense network against oxidative damage.

Sea cucumbers represent a promising source of biomedical and agrochemical products for the treatment and prevention of various diseases [29]. In the seafood industry, a substantial amount of waste is generated during sea cucumber processing, with the viscera often discarded [30]. Traditionally, these by-products have not only contributed to resource wastage but also posed environmental hazards. However, accumulating evidence indicates that sea cucumber viscera are rich in various bioactive compounds, such as polysaccharides, peptides, and saponins, which exhibit significant antioxidant, anti-inflammatory, and immunomodulatory effects [31,32]. For instance, polysaccharides derived from the viscera can effectively scavenge free radicals, while saponins are known to enhance cellular antioxidant enzyme activities [33,34]. Although individual components such as sea cucumber polysaccharides exhibit clear bioactivities, traditional extraction processes are often inefficient, cumbersome and costly, limiting their potential for large-scale applications [35,36]. Therefore, instead of isolating single active ingredients, investigating the holistic biological effects and application potential of sea cucumber viscera—as a complex mixture of multiple functional constituents—may offer a more practical and cost-effective research approach. Despite their promising bioactive potential, the functional utilization of sea cucumber viscera remains at an early exploratory stage, warranting further investigation to realize their full practical value. This study aims to elucidate the antioxidant capacity of sea cucumber viscera (SCV) and its role in modulating gut microbiota using a mouse model of chronic cold-induced oxidative stress. Specifically, we will assess the effects of sea cucumber viscera on oxidative damage, the activation of the Keap1-Nrf2/HO-1 signaling pathway, and the subsequent changes in gut microbiota composition. This research provides a theoretical foundation for using SCV in oxidative stress intervention while promoting the conversion of this agricultural by-product into a high-value functional food.

## 2. Materials and Methods

### 2.1. Material Preparation

Fresh sea cucumber intestines (the primary digestive and absorptive organ of sea cucumbers) and sea cucumber gonads (a portion of the sea cucumber’s reproductive glands) were purchased from Yantai City, Shandong Province, China. First, the sea cucumber intestines and gonads were thoroughly cleaned to remove any sediment, and then excess surface moisture was absorbed using filter paper. Next, the samples were pre-frozen at −80 °C for 12 h. Afterward, the samples were placed in a freeze dryer (Tianjin Port East Technology Development Co., Ltd., Tianjin, China, model: CoolSafeTM) for 24 h of vacuum freeze-drying. Following the freeze-drying process, the sea cucumber intestines and gonad samples were ground and passed through a 40-mesh sieve. They were then mixed evenly in a 1:1 weight ratio to create a freeze-dried viscera powder, which was finally stored at 4 °C for subsequent experiments.

### 2.2. Determination of Nutrient Composition of Sea Cucumber Viscera

The crude protein content was determined following the national standard method GB5009.5-2016 (Kjeldahl method) [37]. Approximately 0.60 g of the sample (recorded as m) was weighed into a digestion tube. Then, 0.40 g of copper sulfate (CuSO_4_), 6.00 g of anhydrous potassium sulfate (K_2_SO_4_), and 10 mL of concentrated sulfuric acid (H_2_SO_4_, 98%) were added. The mixture was digested at 420 °C until it turned transparent with a blue-green color. After cooling, 30 mL of distilled water was added. The digest was then transferred to an automatic Kjeldahl apparatus (FOSS, Beijing, China) for distillation, and the liberated ammonia was absorbed in a boric acid solution. The absorbed ammonia was titrated with a standard hydrochloric acid (HCl) solution (0.1 mol/L). The titration endpoint was determined by a color change in the mixed indicator to a faint pink or light red. The volume of HCl consumed in the sample titration was recorded as V_1_. A blank titration was performed using sucrose instead of the sample, and the volume consumed was recorded as V_2_. The nitrogen-to-protein conversion factor (F) for sea cucumber viscera was taken as 6.25. The crude protein content was calculated using the following formula:

$$\omega (\mathrm{Crude}\;\mathrm{Protein})=\frac{(V1-V2)\times C_{HCL}\times 0.0140\times F}{m}\times 100\%$$

Crude fat content was determined using the Soxhlet extraction method in accordance with the Chinese national standard GBT14772-2008 [38]. The sample was first dried in an oven at 105 °C for 2 h to achieve constant weight, recorded as the initial dry mass (m_0_). Approximately 2.00 g of the dried sample was accurately weighed and wrapped in a fat-free filter paper pouch, with the combined mass recorded as m_1_. The wrapped sample was placed in an extraction tube, and 100 mL of petroleum ether was added to the extraction flask. The extraction was conducted in a water bath maintained at 65 °C, with a reflux frequency of 10 cycles per hour for a total duration of 10 h to achieve approximately 100 complete extraction cycles. Following extraction, the sample was ventilated in a fume hood for 30 min to evaporate residual solvent, followed by drying at 105 °C for 1 h to remove traces of moisture. The sample was then cooled in a desiccator to room temperature and weighed to obtain the final dry mass (m_2_). The crude fat content was calculated as follows:

$$\omega (\mathrm{Crude}\;\mathrm{Fat})=\frac{m1-m2}{m0}\times 100\%$$

The moisture content was determined using the direct drying method specified in the Chinese National Standard GB 5009.3-2016 [39]. An aluminum weighing bottle was cleaned, dried, and weighed to obtain its tare weight (m_0_). Then, 2.00 g of a homogenized sample was accurately weighed into the bottle, and the combined mass (m_1_) was recorded. The sample was placed in a drying oven at 105 °C and dried for 4 h. After drying, the bottle was transferred to a desiccator containing silica gel for 30 min of cooling to prevent moisture absorption. The final mass (m_2_) was then recorded. The moisture content was calculated using the following formula:

$$\omega (\mathrm{Moisture}\;\mathrm{Content})=\frac{m1-m2}{m1-m0}\times 100\%$$

The crude ash content was determined using the dry ashing method in accordance with the Chinese National Standard GB 5009.4-2016 [40]. A pre-cleaned porcelain crucible and its lid were placed in a muffle furnace at 550 ± 25 °C for 30 min until achieving constant weight. After cooling in a desiccator containing silica gel to room temperature (25 °C), the tare weight (m_0_) was recorded. A sample of 3.00 g was accurately weighed into the crucible, and the combined mass (m_1_) was recorded. The sample was then gradually carbonized on an electric hotplate at low temperature until smoke emission ceased. Subsequently, The crucible containing the carbonized sample was transferred to the muffle furnace preheated to 550 °C and ashed for 3 h. After ashing, the crucible was cooled to approximately 200 °C in the furnace doorway, then transferred to a desiccator for 30 min to reach room temperature. The final mass (m_2_) of the crucible and ash was then recorded. The crude ash content was calculated as follows:

$$\omega (\mathrm{Crude}\;\mathrm{Ash})=\frac{m2-m0}{m1}\times 100\%$$

The calcium content was determined using the potassium permanganate (KMnO_4_) titration method in accordance with the Chinese National Standard GBT6436-2018 [41]. To the crucible containing the crude ash sample, 10 mL of hydrochloric acid (HCl, 1:1 *v*/*v*) and a few drops of concentrated nitric acid (HNO_3_, 65–68%) were added. The mixture was heated and boiled gently for 15 min to complete digestion. The digested solution was quantitatively transferred to a 100-mL volumetric flask (V_1_). A 10 mL aliquot (V_2_) of the digest solution was pipetted into a 250-mL beaker. Then, 100 mL of distilled water and 2 drops of methyl red indicator solution (0.1% *w*/*v*) were added. Ammonia solution (10% *w*/*v*) was added dropwise until the color changed from red to orange-yellow. Then, dilute hydrochloric acid (0.1 M) was used to adjust the pH precisely to 2.5–3.0 using a calibrated pH meter. Subsequently, 10 mL of ammonium oxalate solution (5% *w*/*v*) was added. The mixture was heated to boiling, then incubated at 90–95 °C for 12 h (overnight) to facilitate complete precipitation of calcium oxalate. The precipitate and filter paper were quantitatively transferred back to the original beaker. Then, 10 mL of sulfuric acid (H_2_SO_4_, 50% *v*/*v*) and 50 mL of distilled water were added, and the mixture was heated to 70–80 °C to dissolve the precipitate completely. The solution was maintained at 70–80 °C and titrated with 0.05 mol/L standard potassium permanganate (KMnO_4_) solution. The endpoint was reached when a faint pink color persisted for 30 s. The volume of KMnO_4_ consumed was recorded as V_3_. A blank titration was conducted using identical volumes of sulfuric acid and distilled water (without the sample), and the volume of KMnO_4_ consumed was recorded as V_0_ for correction. The calcium content (%, mass fraction) was calculated using the following formula, where mis the mass of the crude ash sample (g), Mis the molar concentration of KMnO_4_ (mol/L), and volumes are in mL:

$$\omega (\mathrm{Calcium}\;\mathrm{content})=\frac{(V3-V0)\times c_{KMnO4}\times 0.02}{m}\times \frac{V1}{V2}\times 100\%$$

The phosphorus content was determined using the molybdenum yellow spectrophotometric method in accordance with the Chinese National Standard GB 5009.87-2016 [42]. A series of phosphorus standard solutions at varying concentrations were prepared by diluting to 50 mL with distilled water, followed by the addition of 10 mL of ammonium vanadate-molybdate color-developing reagent. After thorough vortex mixing, the solutions were allowed to stand for 10 min at room temperature (25 ± 2 °C). The absorbance was then measured at 440 nm using a spectrophotometer to establish a calibration curve. A 10 mL aliquot of the sample digest solution was analyzed identically to measure its absorbance. The phosphorus content was calculated by interpolating the absorbance value onto the calibration curve.

The polysaccharide content was calculated according to the following formula: Polysaccharide (mg/g) = Total Sugar − Reducing Sugar. The total sugar content in sea cucumber viscera was determined using the phenol-sulfuric acid method. Approximately 2.00 g of sample was homogenized with 10.0 mL of distilled water using a mechanical grinder. The homogenate was centrifuged at 10,000× *g* for 15 min at 4 °C, and the supernatant was collected as the sample solution. A glucose stock solution (1000 mg/L) was prepared and serially diluted to concentrations of 20, 40, 60, 80, and 100 mg/L for calibration. For each standard, 1.0 mL of 6% (*w*/*v*) phenol solution was added, followed by rapid vertical addition of 5.0 mL of concentrated sulfuric acid, the final volume was adjusted to 8.0 mL with distilled water. The mixture was vortex-mixed for 30 s and incubated at room temperature (25 ± 2 °C) for 30 min to complete color development. The absorbance was measured at a wavelength of 490 nm to establish a total sugar standard curve. Similarly, 1.0 mL of the sample solution was analyzed in triplicate using the identical phenol-sulfuric acid protocol, and the mean absorbance value was recorded. The total sugar content was calculated by interpolating the sample absorbance on the standard curve and expressed as glucose equivalents per gram of sample (mg/g). The reducing sugar content was determined using the 3,5-dinitrosalicylic acid (DNS) method. A glucose standard solution was prepared by serial dilution to generate a concentration gradient for calibration. To each standard solution, 2 mL of DNS reagent was added, and the volume was adjusted to 4 mL with distilled water. The mixture was heated in a water bath at 90 °C for 5 min to facilitate color development, cooled to room temperature, and diluted to a final volume of 10 mL with distilled water. Absorbance was measured at 540 nm using a spectrophotometer, and a standard curve was plotted by relating absorbance values to known glucose concentrations. Following the identical DNS protocol, 1 mL of the sample solution was processed, and its absorbance was measured under the same conditions. The reducing sugar concentration was calculated by interpolating the sample absorbance onto the standard curve. All measurements were conducted using a microplate reader (Molecular Devices, CMax Plus, Shanghai, China).

Fatty Acid Content was determined by the internal standard method using a 7890B gas chromatograph (Agilent Technologies, Santa Clara, CA, USA). The basic reaction conditions for gas chromatography include a DB-WAX gas capillary column, nitrogen as the carrier gas, a split ratio of 10:1, and an ion flame detector, with inlet and detector temperatures set at 250 °C.

### 2.3. Determination of Antioxidant Capacity of Sea Cucumber Viscera In Vitro

Fresh sea cucumber viscera were accurately weighed and homogenized with an equal volume of distilled water (1:1 *w*/*v*) using a mechanical homogenizer to obtain a uniform slurry. Subsequently, the homogenate was serially diluted in phosphate-buffered saline (PBS, pH 7.4) to final concentrations of 0, 20, 40, 60, 80, and 100 mg/mL for subsequent DPPH, OH^−^, and O_2_^−^ radical scavenging assays. Similarly, lyophilized powder of sea cucumber viscera was reconstituted in distilled water (1:1 *w*/*v*) and vortex-mixed to homogeneity. The resulting suspension was diluted with PBS to prepare concentration gradients of 0, 2, 4, 6, 8, and 10 mg/mL for the DPPH and OH^−^ scavenging assays, and 0, 2, 4, 6, 8, 10, and 12 mg/mL for the O_2_^−^ scavenging assay. The in vitro antioxidant capacity was evaluated using commercial assay kits (Nanjing Jiancheng Bioengineering Institute, Nanjing, China) for DPPH, hydroxyl radical (OH^−^), and superoxide anion radical (O_2_^−^) scavenging, strictly following the manufacturer’s instructions.

### 2.4. Animal Experiments

The Animal Ethics Committee of Tianjin Agricultural University reviewed and approved animal experiments on chronic cold stress and sea cucumber viscera gavage in accordance with the National Research Council’s Guidelines for the Care and Use of Laboratory Animals (Approval number: 2023LLSC26).

Thirty-five SPF-grade KM mice, 7 weeks old, female, weighing 27–30 g, were purchased from Beijing Vital River Experimental Animal Technology Co., Ltd. (in Beijing, China), and were fed a diet provided by the same company. The feeding environment was maintained at a temperature of 23–25 °C, with humidity between 40% and 50%, and a light–dark cycle of 12 h each. Mice had free access to food and sterile water. The animal experiment lasted for 28 days, during which the first 1–3 days were allocated for adaptive feeding. From day 4 to day 7, an adaptation period for gastric lavage was conducted. During this period, all mice received a daily gastric lavage with the corresponding substance: the control group and CS group received 0.9% saline solution, while the SCV group received their corresponding dose of freeze-dried sea cucumber viscera powder (dissolved in saline solution) to acclimate them to the gastric lavage procedure. From days 7 to 28 (formal experimental period), all treatment groups, except for the control group, were subjected to cold stimulation for 3 h daily at 4 °C to establish a chronic cold stress mice model. The experimental design is grouped as in Table 1.

Sea cucumber visceral freeze-dried powder was added to 0.9% normal saline according to varying doses for different treatment groups and administered daily via intragastric feeding before the onset of cold stress. The control group and the cold stress (CS) group received intragastric administration of 0.9% normal saline. Mice were randomly divided into 5 groups (7 mice per group) based on the treatment method, which included Control, CS (Cold stress), SCV-L (SCV 200 mg/kg/d + CS), SCV-M (400 mg/kg/d + CS) and SCV-H (800 mg/kg/d + CS). On the 28th day, 24 h after the last gavage, blood was collected following a 12 h fasting period. Blood samples were centrifuged at 4000 rpm for 10 min to separate the serum. Tissues were collected, weighed, frozen in liquid nitrogen, and stored at −80 °C.

### 2.5. Growth Performance and Rectal Temperature Measurement

Body weight, feed intake, and rectal temperature of mice were recorded every 3 days during the experiment, with rectal temperature measured before and after cold stress on the last day. Average daily gain (ADG) and average feed intake (ADFI) were calculated following the experiment.

### 2.6. Biological Index Detection

The organ index was calculated as the ratio of tissue weight to body weight [organ index = organ weight (g)/body weight (g) × 100%]. Serum and liver samples collected during dissection were homogenized in a low-temperature tissue grinder for 1 min, centrifuged for 10 min at 12,000 r/min, and supernatant was taken to obtain the test product. The levels of malondialdehyde (MDA), total superoxide dismutase (T-SOD), glutathione peroxidase (GSH-Px), aspartate aminotransferase (AST), alkaline phosphatase (ALP), and alanine aminotransferase (ALT) in the liver and serum were determined using a commercial kit (Nanjing Jiancheng Bioengineering Institute, Nanjing, China). All measurement steps were performed in accordance with the manufacturer’s instructions.

### 2.7. Histopathological Assessment

Fresh mice livers were rinsed three times with pre-cooled 0.9% normal saline and fixed in 4% paraformaldehyde for 24 h. Hematoxylin-eosin staining (HE) staining was employed to make liver histopathologic sections for general examination. A histopathological score was assigned, and the specific scores are shown in Table 2.

### 2.8. Determination of Cold Stress-Related Genes in Liver by Real-Time Quantitative PCR

Real-time fluorescence quantitative PCR (RT-qPCR) was used to detect cold stress-related genes HSP70, HSP90, Keap1, Nrf2, and HO-1 in the liver. Total RNA was extracted from tissues using Trizol (Thermo Fisher Scientific, Carlsbad, CA, USA). The cDNA was synthesized using the RevertAid First Strand cDNA Synthesis Kit (Thermo Fisher Scientific, Carlsbad, CA, USA). Step One Plus™ Real-Time PCR System (Applied Biosystems, Carlsbad, CA, USA) was used to determine the mRNA expression levels. The primer sequences are provided in Supplemental Table S1 (with GAPDH as the reference gene). The 2^−^^ΔΔCT^ method was used to calculate the mRNA relative expression, three replicates in each group.

### 2.9. Gut Microbiota Analysis

On the 28th day, mice were executed, the abdominal cavity was opened in a sterile environment, the cecum was cut longitudinally, and fresh feces were collected, placed in sterile, enzyme-free tubes, and stored at −80 °C. Genomic DNA was extracted from fecal samples using a commercial DNA extraction kit (Thermo Fisher Scientific, Carlsbad, CA, USA). DNA concentration was quantified using a Nanodrop spectrophotometer, and integrity was verified by 1.2% agarose gel electrophoresis. The V3-V4 hypervariable region of the 16S rRNA gene was amplified with barcoded primers (forward: 5′-ACTCCTACGGGAGGCAGCA-3′; reverse: 5′-GGACTACHVGGGTWTCTAAT-3′) using a standardized DNA template. PCR amplicons were purified with Vazyme VAHTS Magnetic Beads and quantified via the Quant-iT PicoGreen dsDNA Assay Kit (Invitrogen, Carlsbad, CA, USA).

The purified amplicons were pooled, quantified, and homogenized to construct a sequencing library. Paired-end sequencing (2 × 250 bp) was performed on the Illumina NovaSeq 6000 platform using the SP Reagent Kit (both are from Illumina, Shanghai, China). Raw sequencing reads were demultiplexed with the QIIME2 demux plugin, and adapter sequences were trimmed with cutadapt. The DADA2 pipeline was applied for quality control, denoising, read merging, and chimera removal, producing amplicon sequence variants (ASVs) and an abundance table at 100% sequence similarity. ASVs were taxonomically annotated using the Greengenes database. Low-abundance ASVs (<0.001% of total reads) were filtered out, generating a final abundance matrix for downstream analysis. Bar plots visualizing ASV counts and taxonomic distributions across samples were generated using R software (version 4.0.0). Alpha diversity indices, beta diversity, and community composition were analyzed using QIIME2 (version 2019.4) and specific R packages (version 4.0.0). Seven fecal samples per group were randomly selected for gut microbiota profiling. DNA extraction and 16S rRNA sequencing were performed by Shanghai Personal Biotechnology Co., Ltd. (in Shanghai, China).

### 2.10. Statistics Analysis

In this study, the determination of nutrient composition and in vitro antioxidant effects of sea cucumber viscera were statistically analyzed using GraphPad Prism 9.5 software; the experiments on the effects of sea cucumber viscera on chronic cold stress in mice were analyzed by one-way ANOVA using SPSS 25.0 software, and post hoc multiple comparisons conducted using the Duncan’s and Tukey test. Plotting images using Origin 2024 and GraphPad Prism 9.5 software. The experiments on the effects of sea cucumber viscera on the gut microbiota of mice experiencing chronic cold stress were analyzed using the Parsonage Genomic Cloud online platform (https://www.genescloud.cn/hom, accessed on 29 September 2023) for data analysis and plotting. Statistical significance was defined as (*) or (#) when *p* < 0.05.

## 3. Results

### 3.1. Nutritional and Antioxidant Properties of Sea Cucumber Viscera

**Figure 1:**
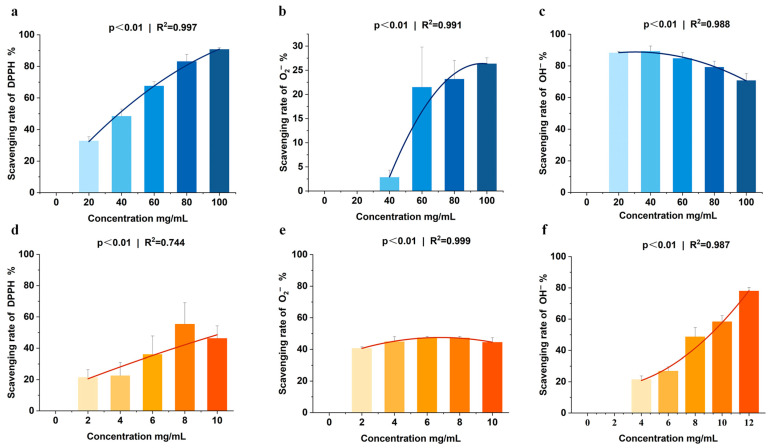
(**a**–**c**). In vitro antioxidant activity of sea cucumber viscera homogenate and its lyophilized powder. (**a**–**c**) Scavenging activities of sea cucumber viscera homogenate on three oxidizing free radicals: (**a**) DPPH, (**b**) superoxide anion (O_2_^−^), and (**c**) hydroxyl radical (OH^−^). (**d**–**f**) Scavenging activities of lyophilized sea cucumber viscera powder on: (**d**) DPPH, (**e**) O_2_^−^, and (**f**) OH^−^. Data are presented as the mean ± SD (*n* = 3).

Sea cucumber viscera are rich in nutrients, and the natural antioxidant compounds—such as polysaccharides, peptides, and saponins—have been shown to protect animal tissues and organs from oxidative damage caused by free radicals. In this study, sea cucumber viscera, including intestines and gonads, underwent freeze-drying to analyze their main nutrients: crude protein (56.97%), crude fat (24.27%), and crude ash (7.47%). Sea cucumber viscera polysaccharides with good antioxidant activity were measured at 4.76% (Table 3). Fatty acid profiling demonstrated that sea cucumber viscera (SCV) contained 26 identified fatty acids, with unsaturated fatty acids (UFA) representing 63.72% of the total lipid content. Specifically, monounsaturated fatty acids (MUFA) accounted for 35.93%, while polyunsaturated fatty acids (PUFA) constituted 27.79%. The major MUFAs included oleic acid (C18:1), palmitoleic acid (C16:1), and myristoleic acid (C14:1). Notable PUFAs comprised gamma-linolenic acid (C18:3γ), docosatrienoic acid (C20:3), arachidonic acid (C20:4), and docosahexaenoic acid (C20:5) (Table 4). Additionally, sea cucumber viscera exhibited a significant scavenging effect on the three in vitro free radicals, DPPH, O_2_^−^ and OH^−^. In the concentration range of 0–100 mg/mL, the scavenging rate of DPPH and O_2_^−^ increased with higher sample concentrations, demonstrating a pronounced scavenging effect on OH^−^ as well (Figure 1a–c). The clearance of OH^−^ by the lyophilized powder of sea cucumber viscera within the 0–12 mg/mL concentration range showed a positive correlation with sample concentration. The lyophilized powder also exhibited enhanced scavenging activity against DPPH and O_2_^−^ (Figure 1d–f). Notably, the DPPH radical scavenging curve of the freeze-dried powder reaches a plateau at higher concentrations, deviating from strict dose-dependency (Figure 1d). This deviation likely arises from the complex composition of sea cucumber viscera, in which multiple antioxidant components exhibit reaction saturation at elevated concentrations. These results indicate that sea cucumber viscera not only have nutritional value but also have antioxidant properties in vitro.

### 3.2. Sea Cucumber Viscera Ameliorate Oxidative Stress Induced Organ Damage in Mice

**Figure 2:**
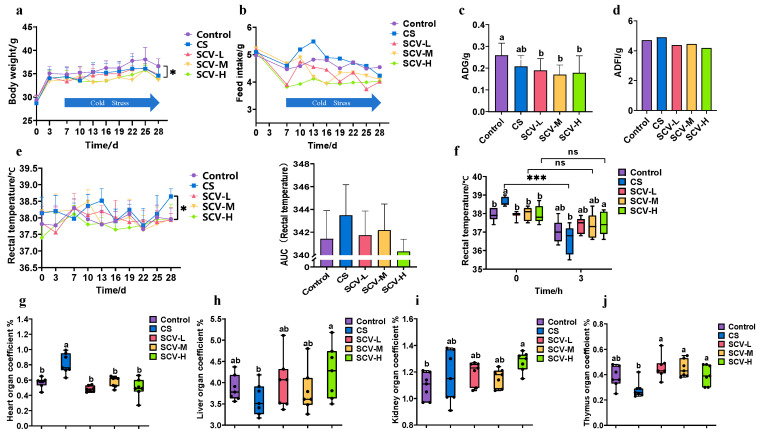
Effect of sea cucumber on growth performance and organ indices of cold-stressed mice (**a**) Body weight. (**b**) Feed intake. (**c**) Mean daily weight gain. (**d**) Mean daily feed intake. (**e**) Rectal temperatures during cold stress. (**f**) Rectal temperatures on day 28 before and after cold stress. (**g**) heart index. (**h**) Liver index. (**i**) Kidney index. (**j**) Thymus index. Results are the mean ± SD (*n* = 7). * *p* < 0.05 and *** *p* < 0.001 vs. Control group. Different letters indicate the significant difference among the treatments (*p* < 0.05).

On the 28th day of the experiment, both the CS group and the SCV group at each dose exhibited a significant decrease in body weight (*p* < 0.05) and a declining trend in food intake compared to the control group (*p* > 0.05) (Figure 2a,b). The average daily weight gain of mice in the CS group showed a decreasing trend, while the average daily food intake demonstrated an increasing trend compared to the control group (Figure 2c,d), these findings are consistent with the results of previous studies on chronic cold stress [43]. The mean daily weight gain was significantly lower (*p* < 0.05) and average daily food intake tended to decrease in all dose groups compared to the control group (Figure 2c,d). On the 28th day of the experiment, rectal temperature was significantly higher in the CS group compared to the control group; however, no significant differences were observed between the dose groups and the control group (Figure 2d). When comparing rectal temperatures before and after the 28th day of stress, the temperature was significantly lower in the CS group after stress (*p* < 0.001). In contrast, no significant changes were noted in the temperatures before and after stress in each dose group. Before cold stress, rectal temperatures were significantly higher in the CS group compared to each dose group (*p* < 0.05), while the temperatures in the SCV-H group were significantly higher than those in the CS group after cold stress (*p* < 0.05) (Figure 2f). These results indicate that sea cucumber viscera possess a notable ability to maintain body temperature and resist cold.

The viscera index provides a visual measure of the health status of the animal organism. Comparing the cardiac indices across groups revealed a significant increase in the CS group (*p* < 0.05), this CS-induced increase was significantly reduced in all dose groups of SCV (*p* < 0.05) (Figure 2g). Liver indices in the SCV-H group were significantly higher than those in the CS group (*p* < 0.05) (Figure 2h); Similarly, kidney indices in the SCV-H group were significantly higher than those in the control group (Figure 2i). Furthermore, the thymus index in each dose group of SCV was significantly higher than that in the CS group (*p* < 0.05) (Figure 2j).

### 3.3. Sea Cucumber Viscera Have Antioxidant Capacity

**Figure 3:**
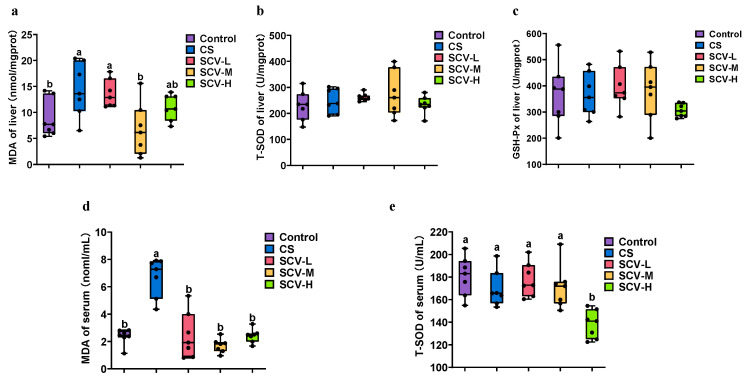
Effects of cold stress on oxidation and antioxidant enzymes in liver and serum of mice. (**a**–**c**) Determination of MDA, T-SOD, and GSH-Px enzyme activity in liver. (**d**,**e**). Detection of MDA, T-SOD and enzyme activity in serum. Results are the mean ± SD (*n* = 7). Different letters indicate the significant difference among the treatments (*p* < 0.05).

Oxidative stress disrupts the animal body’s redox system, as excess ROS interferes with the body’s antioxidant defense system, resulting in increased MDA levels and decreased T-SOD and GSH-Px activities. This imbalance can damage tissues and organs. In the analysis of liver antioxidant enzyme activities, MDA levels in both liver and serum were significantly higher in the CS group compared to the control group (*p* < 0.05). However, MDA activity was significantly reduced in all SCV dose groups relative to the CS group, with SCV-M notably decreasing MDA activity in both liver and serum (*p* < 0.05) (Figure 3a,d). No significant differences in T-SOD and GSH-Px activities were observed in the liver across groups (Figure 3b,c) though T-SOD activity in serum was significantly decreased in the SCV-H group (*p* < 0.05). These findings indicate that sea cucumber viscera may possess antioxidative properties that mitigate oxidative damage.

### 3.4. Sea Cucumber Viscera Can Alleviate Oxidative Stress Induced Liver Impairment

**Figure 4:**
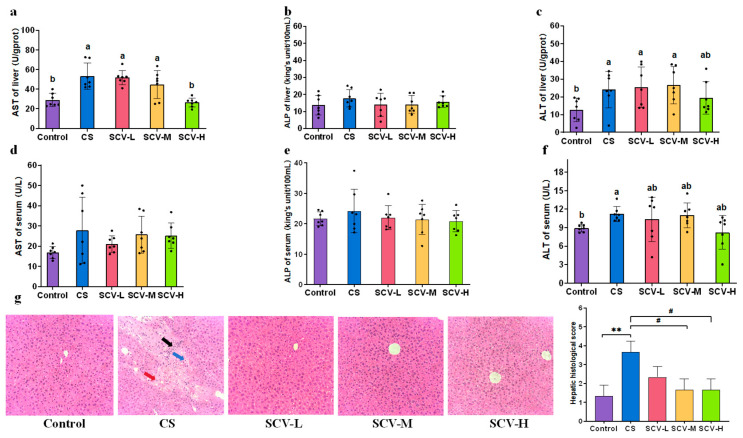
Protective effect of sea cucumber viscera on liver. (**a**–**c**) AST, ALP, ALT of liver. (**d**–**f**) AST, ALP, ALT of serum. (**g**) H&E staining of the liver (200× magnification). Red arrows: erythrocyte exudate; Black arrows: Kupffer cell exudate; Blue arrows: lymphocyte exudate. Results are the mean ± SD (*n* = 7). Different letters indicate the significant difference among the treatments (*p* < 0.05). ^#^ *p* < 0.05 vs. CS, ** *p *< 0.01 vs. Control.

Liver functional enzymes are indicative of the liver’s physiological functions. In this experiment, we assessed the effects of sea cucumber viscera on liver functional enzymes by measuring AST, ALT, and ALP levels in both serum and liver. The CS group showed significantly elevated liver activities of AST and ALT compared to the control group (*p* < 0.05), with ALP also displaying an upward trend (Figure 4a–c). This suggests that stress inflicts damage to the mice liver. Each SCV dose inhibited liver AST, ALP, and ALT activities, with the SCV-H group significantly reducing AST activity (*p* < 0.05) (Figure 4a–c). The CS group significantly elevated serum ALT activity (*p* < 0.05), with AST and ALP showing an upward trend (Figure 4d–f). The SCV dose groups exhibited inhibitory effects on serum AST, ALP, and ALT activities (Figure 4d–f).

Pathologic histological analysis of the liver was conducted to evaluate the protective effect of sea cucumber viscera. Liver tissue from control mice was well-structured, with clear nuclei and cytoplasm and no evident lesions. In contrast, CS mice displayed enlarged hepatocytes, with some hepatocyte nuclei fragmented or even disappeared, disorganized arrangement of hepatocyte cords, disappearance of interstitial space, and exudation of a large number of Kupffer’s cells with erythrocyte and lymphocyte infiltration. This confirms that chronic cold stress induces liver injury in mice. Observations of liver sections from each dose of the SCV group revealed reduced intracellular vacuoles, clearer liver cells, and decreased exudation of Kupffer cells and erythrocytes (Figure 4g). Collectively, these data suggest that sea cucumber viscera may mitigate oxidative stress induced liver injury.

### 3.5. Sea Cucumber Viscera Alleviate Oxidative Stress by Modulating the Keap1-Nrf2/HO-1 Signaling Pathway

**Figure 5:**
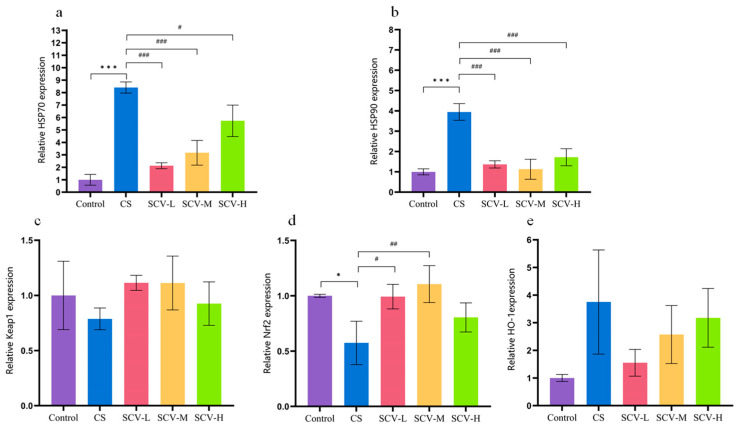
Effects of sea cucumber viscera supplementation on the liver gene expression of cold stressed mice. (**a**) HSP70. (**b**) HSP90. (**c**) Keap1. (**d**) Nrf2. (**e**) HO-1. Results are the mean ± SD (*n* = 7). * *p* < 0.05  and *** *p* < 0.001 vs. Control. ^#^ *p* < 0.05, ^##^ *p* < 0.01 and ^###^ *p* < 0.001 vs. CS.

To investigate the mechanism by which sea cucumber viscera alleviates cold stress-induced oxidative stress, the mRNA expression levels of HSP70, HSP90, Keap1, Nrf2, and HO-1 in the liver were measured by RT-qPCR. Compared with the control group, mice in the cold stress (CS) group exhibited significantly elevated mRNA expression of HSP70 and HSP90 (*p* < 0.001), Concurrently, Nrf2 mRNA expression was significantly suppressed (*p* < 0.05), while Keap1 expression showed a decreasing trend and HO-1 expression an increasing trend, suggesting a disrupted antioxidant defense system under cold stress. SCV administration effectively counteracted these changes. Specifically, the low- and medium-dose SCV groups (SCV-L, SCV-M) significantly reduced the overexpression of HSP70 and HSP90 (*p* < 0.001) and markedly up-regulated Nrf2 mRNA expression (*p* < 0.05 and *p* < 0.01, respectively) compared to the CS group. Keap1 mRNA levels also showed a tendency to increase, while HO-1 mRNA exhibited a downward trend. The high-dose group (SCV-H) significantly decreased HSP70 and HSP90 expression (*p* < 0.05 and *p* < 0.001, respectively), with non-significant increasing trends observed for Nrf2 and Keap1 mRNA and a decreasing trend for HO-1 mRNA (Figure 5a–e). These results suggest that SCV mitigates cold stress-induced liver injury by modulating the heat shock response and activating the Keap1-Nrf2-HO-1 signaling pathway, thereby enhancing the endogenous antioxidant defense system.

### 3.6. Sea Cucumber Viscera Alters the Gut Microbial Community in Oxidative Stress Mice

**Figure 6:**
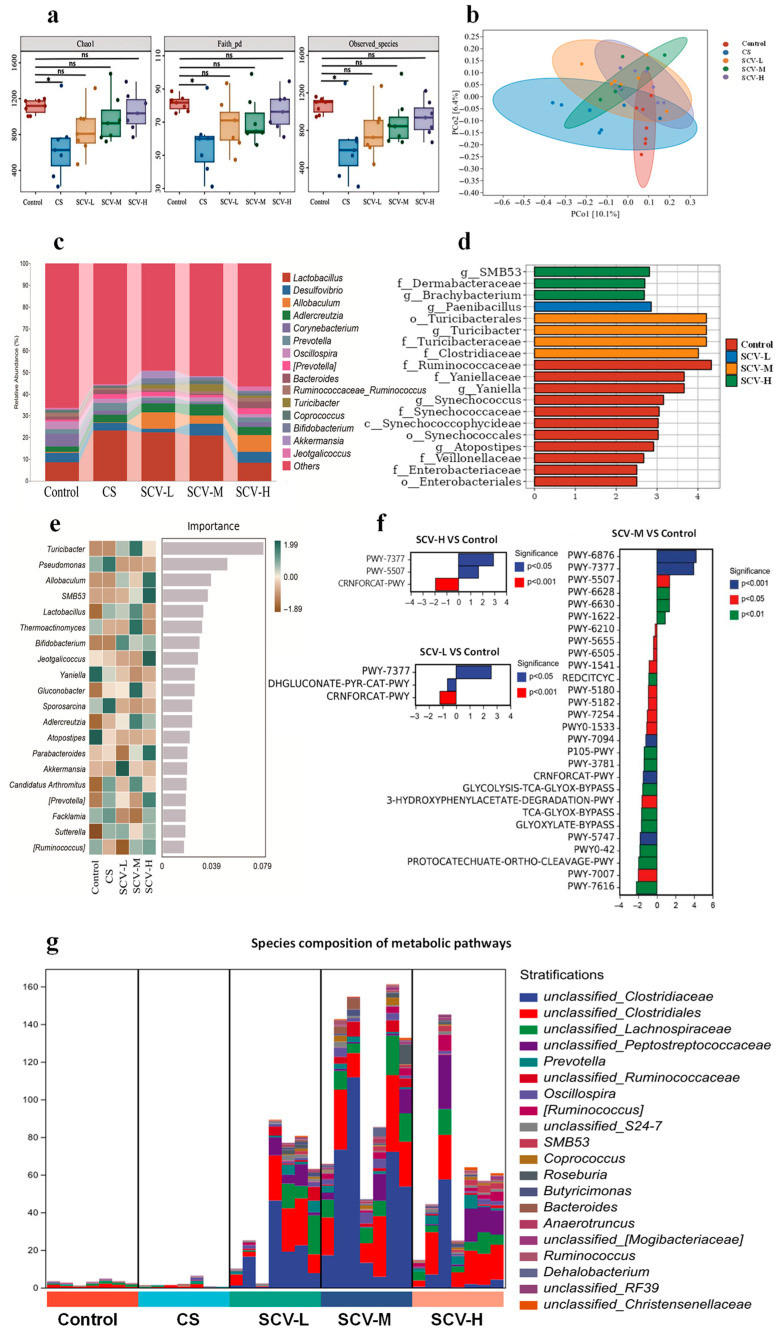
Effect of sea cucumber viscera on gut microbiota of mice. The feces of experimental mice were subjected to 16S rRNA sequencing. (**a**) α-Diversity indices, i.e., Chao index, Faith_pd index, and Observed_species, represent the abundance and diversity of the groups. (**b**) PCoA analysis based on the Bray–Curtis distance shows the β-diversity of each community at the OTU level. (**c**) Genus level bacterial taxonomic distribution of communities (top 15 in abundance). (**d**) LDA histograms from LEFSe analysis showing different gut microbiota enriched in comparison to controls with LDA score > 2. (**e**) Random forest analysis screening the top 20 gut microorganisms (at the genus level) in terms of differences between groups. (**f**) Differential tertiary metabolic pathways for SCV-L/SCV-M/SCV-H VS. Control. (**g**) PWY-7377 pathway colony contribution. Results are the mean ± SD (*n* = 7). * *p* < 0.05 vs. Control.

The effects of sea cucumber viscera on the gut microbiota of mice were evaluated by analyzing the gut microbiota. α-diversity analysis revealed that the Chao1, Faith_pd, and Observed_species indices in the CS group were significantly lower than those of the control group at the OTU level (*p* < 0.05); Chao1, Faith_pd, and Observed_species indices of each dose of SCV group were not significantly different from the control group (Figure 6a). β-diversity analysis showed a separation of microbial structures between the CS group and the SCV group at all doses (Figure 6b). Venn diagram results indicated that the number of OTUs in the CS group was significantly lower than in the control group, while OTU counts in the SCV groups increased in a dose-dependent manner (Supplementary Figure S1a). At the genus level, each dose of sea cucumber viscera altered the composition of species in mice exposed to chronic cold stress. The SCV groups exhibited increased relative abundances of *Allobaculum*, *Turicibacter*, *Bifidobacterium*, and *Akkermansia* compared to the control group (Figure 6c; Supplementary Table S2). The LEfSe analysis further examined the effects of sea cucumber viscera on gut microbiota in oxidative-stressed mice, revealing that the SCV-M group was enriched with the highest number of differential strains and displayed the highest LDA score for *Turicibacter* spp. (Figure 6d). Random forest analysis identified *Turicibacter*, *Allobaculum*, and *Lactobacillus* as the top genera by effect size, with *Turicibacter* showing the highest effect value (Figure 6e). *Turicibacter*, a genus with protective effects on animal health, was thus identified as a key genus for alleviating oxidative stress through sea cucumber viscera, with the SCV-M group demonstrating the most pronounced effects. Furthermore, functional prediction analysis of gut microbiota showed that the SCV-M group had the highest number of differential metabolic pathways, with the metabolic pathway PWY-7377, which is related to vitamin B12 synthesis, showing a highly significant difference compared to the control group (Figure 6f). Based on the differential metabolic pathway results, the top 15 genera with the highest contribution to the PWY-7377 (cob (II) yrinate a, c- diamide biosynthesis I (early cobalt insertion)) passages were further screened as *Unclassified_Clostridiaceae*, *Unclassified_Clostridium*, *Unclassified_Costreptococcaceae*, *Unclassified_Clostridium pullulans*, *Unclassified*_ *Peptostreptococcaceae*, *Prevotella*, *Unclassified_Ruminococcaceae*, *Oscillospira*, *[Ruminococcus]*, *Unclassified_S24-.7*, *SMB53*, *Coprococcus*, *Roseburia*, *Butyricimonas*, *Bacteroides*, *Anaerotruncus* (Figure 6g).

### 3.7. Analysis of the Correlation Between Gut Microbiota and Traits in Mice

**Figure 7:**
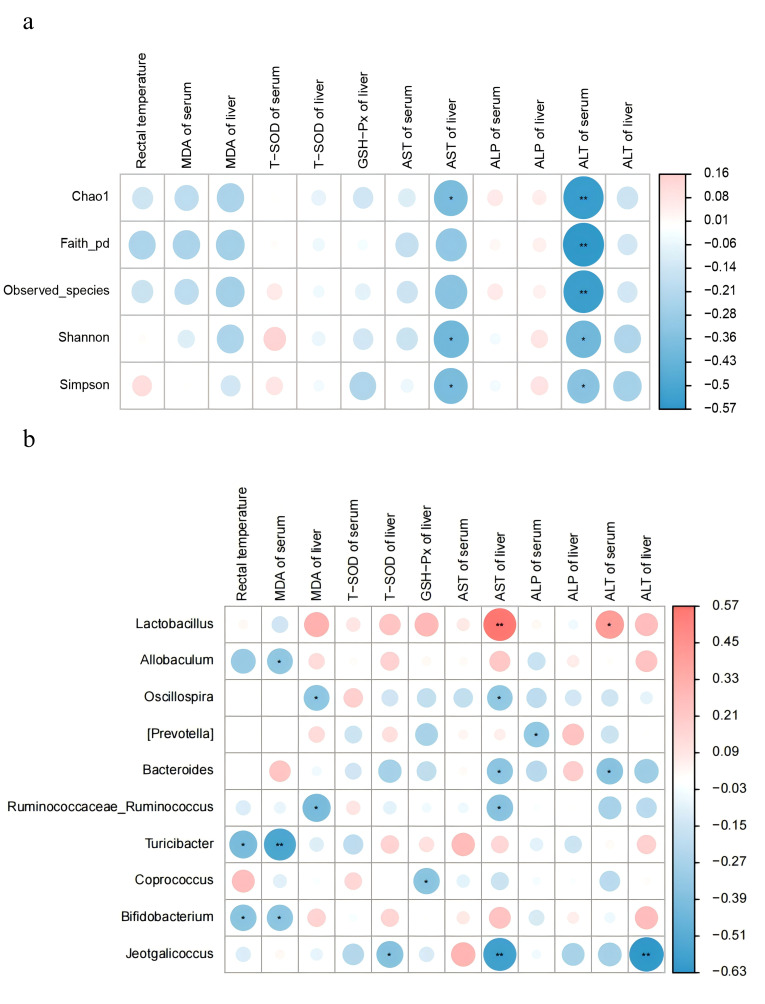
Correlation analysis of gut microbiota with shape in mice. Colors range from blue to red, indicating a change in correlation from negative to positive. (**a**) Correlation analysis of gut microbiota diversity with body temperature, antioxidant enzymes, and hepatic function enzymes. (**b**) Correlation analysis of gut microbiota at the genus level (top 15 in abundance) with body temperature, antioxidant enzymes, and hepatic function enzymes. Results are the mean ± SD (*n* = 7). * *p* < 0.05, ** *p* < 0.01.

Correlation analyses were conducted to further explore the association between gut microbiota and oxidative stress-related indices in mice. The Chao1, Shannon, and Simpson indices were showed a negative correlation with hepatic AST; While the Chao1, Faith_pd, Observed_species, Shannon, and Simpson indices were negatively correlated with serum ALT (Figure 7a). *Allobaculum*, *Turicibacter*, and *Bifidobacterium* were negatively correlated with rectal temperature and serum MDA level. *Ruminococcaceae_Ruminococcus*, and *Oscillospira* were negatively correlated with MDA and AST in liver. In contrast, *Lactobacillus* was positively correlated with AST in liver and ALT in serum (Figure 7b).

## 4. Discussion

During oxidative stress, excess free radicals disrupt the organism’s oxidative-antioxidant balance, causing damage to cellular organelles and physiological functions [44]. Sea cucumber viscera are rich in nutrients, and natural antioxidant compounds such as polysaccharides, peptides, and saponins found in sea cucumber viscera have been shown to protect animal tissues and organs from free radical damage [45,46,47]. Additionally, functional amino acids, including aspartic acid, cysteine, glutamic acid, methionine, arginine, and tryptophan, have demonstrated antioxidant properties [48,49,50,51,52]. Our analysis revealed that sea cucumber viscera contain a variety of antioxidant compounds and amino acids. In vitro antioxidant tests further confirmed the scavenging ability of sea cucumber viscera against free radicals, indicating their potential as an anti-cold stress feed supplement for animals. These findings provide a solid theoretical foundation for subsequent studies using chronic cold stress mice models.

Chronic cold stress disrupts mitochondrial metabolism, leading to excessive ROS production and subsequent oxidative stress [53]. Under these conditions, the activity of key antioxidant enzymes—total superoxide dismutase (T-SOD), the primary enzyme responsible for superoxide radical scavenging, and glutathione peroxidase (GSH-Px), which reduces hydrogen peroxide and lipid peroxides—is often suppressed [54,55]. The accumulation of ROS promotes lipid peroxidation of polyunsaturated fatty acids in cell membranes, resulting in the generation of malondialdehyde (MDA), a recognized biomarker of oxidative damage whose levels are positively correlated with the severity of cellular injury [56]. Consistent with previous reports, hypothermia has been shown to elevate MDA content in plasma and liver tissue while significantly decreasing GSH levels and SOD activity in serum and liver [57,58]. In the present study, cold-stressed mice exhibited significantly increased MDA levels in both liver and serum compared to the control group. SCV intervention, however, significantly counteracted this increase, effectively lowering MDA concentrations. Furthermore, histopathological examination of liver tissue revealed structural alterations such as vacuolar degeneration and inflammatory cell infiltration, providing morphological evidence of oxidative injury and supporting the protective role of SCV in alleviating cold stress-induced hepatic damage.

Fatty acids serve as a critical energy source through mitochondrial β-oxidation, a process that contributes to thermogenesis and helps protect the organism against cold stress [59]. Following intestinal absorption and systemic transport, fatty acids enter the mitochondrial matrix via the carnitine shuttle system. Through a cyclic sequence of dehydrogenation, hydration, re-dehydrogenation, and thiolysis, long-chain fatty acids are progressively cleaved into acetyl-CoA, NADH, and FADH_2_. These metabolites subsequently feed into the tricarboxylic acid cycle and oxidative phosphorylation, efficiently generating ATP for cellular functions while releasing heat [60,61]. In the present study, sea cucumber viscera (SCV) were found to contain 24.27% crude fat, of which unsaturated fatty acids (UFAs) accounted for 63.72%—including 35.93% monounsaturated (MUFAs) and 27.79% polyunsaturated fatty acids (PUFAs). Under cold stress, the elevated energy demand may be partly met through β-oxidation of these dietary UFAs, supporting thermoregulation and overall energy homeostasis—a plausible mechanism for the observed improvement in body temperature maintenance in SCV-fed mice.

Furthermore, PUFAs have been widely reported to counteract oxidative stress by activating the Nrf2 signaling pathway [62]. Certain PUFAs can be metabolized into electrophilic reactive lipid species (RLS) that selectively modify nucleophilic residues on target proteins, thereby regulating Nrf2 activity and mitigating oxidative injury [63]. For instance, oxidized derivatives of omega-3 PUFAs covalently modify Keap1, leading to Nrf2 activation and antioxidant gene expression [64].Similarly, α-linolenic acid and γ-linolenic acid have been shown to alleviate oxidative stress via the Keap1/Nrf2/ARE pathway, underscoring the role of PUFAs in modulating cellular antioxidant defenses [65,66]. The Keap1-Nrf2/HO-1 axis represents a central regulatory mechanism for hepatoprotection under oxidative conditions [67]. Under chronic cold stress, Nrf2 dissociates from Keap1, translocates to the nucleus, and binds to antioxidant response elements (AREs), inducing the expression of cytoprotective genes such as HO-1, which facilitates ROS clearance and alleviates oxidative damage [68,69,70]. In this study, cold stress suppressed hepatic Keap1 and Nrf2 mRNA expression, whereas dietary supplementation with SCV—particularly at the medium dose (SCV-M)—significantly restored Nrf2 expression and normalized Keap1 and HO-1 levels. These results suggest that the activation of the Keap1/Nrf2-HO-1 pathway by SCV may be attributed, at least in part, to its high PUFA content.

This study found that SCV increased the relative abundance of *Turicibacter*, *Akkermansia*, *Allobaculum*, and *Bifidobacterium* in the intestinal microbiota of mice. Among these, *Akkermansia* and *Allobaculum* are known to regulate the thickness of the intestinal mucosal barrier and maintain its integrity [70,71]. *Turicibacter* plays a critical role in protecting host health [72]. *Bifidobacterium* is a major component of the intestinal mucosal biological barrier and is essential for maintaining its integrity [73]. These findings suggest that SCV positively modulates gut microbial diversity and supports intestinal barrier function. Notably, the SCV-M group exhibited the most pronounced effects.

Correlation analysis further revealed that SCV intervention not only significantly increased the relative abundance of *Turicibacter*, *Allobaculum*, and *Bifidobacterium* in the gut, but also demonstrated a negative correlation with serum MDA levels and a positive correlation with hepatic antioxidant enzyme activity. This result suggests that the modulation of gut microbiota may be a key mechanism by which SCV exerts its antioxidant effects. Notably, *Allobaculum* has been shown to enhance the integrity of the intestinal mucosal layer and reduce intestinal permeability, thereby decreasing the translocation of endogenous LPS, which positively impacts gut health [71]. Meanwhile, *Bifidobacterium* can activate the Nrf2 pathway, alleviating liver injury and enhancing the host’s antioxidant defense capacity [74]. Additionally, Ke et al. demonstrated that activation of the Keap1-Nrf2/HO-1 pathway inhibits hepatic inflammation [75]. In this study, oxidative stress induced the infiltration of Kupffer cells in the liver, along with erythrocyte and lymphocyte accumulation. SCV treatment markedly reduced inflammatory infiltration in hepatic tissue. Therefore, we hypothesize that SCV may alleviates hepatic oxidative damage by enhancing the abundance of beneficial gut bacteria, maintaining the intestinal barrier, and synergistically activating the Keap1-Nrf2/HO-1 pathway. This multi-target regulatory mechanism offers novel insights into the antioxidant effects of SCV and preliminarily confirms the central role of gut microbiota–host interactions in stress adaptation. Future studies should employ multi-omics approaches to elucidate how SCV influences the composition of gut microbiota and its metabolites, thereby revealing the specific pathways involved in the antioxidant process. It should be noted that the freeze-dried powder of sea cucumber viscera used in this study was not subjected to sterilization and therefore retains its autochthonous microbiota. Although the total bacterial count (2 × 10^3^ CFU/100 mg) is negligible compared to the host’s established gut microbiome and unlikely to support substantial colonization, it is prudent to acknowledge that microbial metabolites derived from this community may still exert indirect effects on gut microbiota composition and host physiology. Future investigations comparing sterilized versus non-sterilized preparations would help delineate the specific contributions of microbial components from those of the host-derived bioactive factors. Although our findings establish a theoretical foundation for the antioxidant potential and gut microbiota-modulating effects of sea cucumber viscera (SCV), the translation of SCV into functional foods necessitates rigorous safety evaluation. It is essential to verify that these promising natural resources are free from hazardous contaminants, including pathogens, heavy metals, and other toxic compounds that could compromise consumer safety. Therefore, future research should prioritize systematic safety assessments of SCV. Such rigorous safety validation will provide critical data for evaluating SCV’s suitability as a dietary supplement, facilitating its regulatory approval and sustainable application in the functional food industry.

## 5. Conclusions

This research confirms that sea cucumber viscera are rich in nutrients and possess antioxidant and anti-stress properties. Sea cucumber viscera can improve the body temperature maintenance, regulate the levels of oxidative and antioxidant enzymes, attenuate oxidative stress-induced liver injury, and protect the liver by activating the Keap1-Nrf2/HO-1 pathway in mice. The SCV-M group (400 mg/kg/d) exhibited the most significant effects. These findings reveal a gut–liver axis mechanism underlying the antioxidant effects of SCV, highlighting its potential as a promising functional food. Future studies should further elucidate microbial–metabolic interactions to facilitate clinical translation.

## Figures and Tables

**Table 1:** Experimental design grouping.

Group	Experimental Treatment
Control	0.9% Normal saline
CS	0.9% Normal saline
SCV-L	200 mg/kg/d + CS
SCV-M	400 mg/kg/d + CS
SCV-H	800 mg/kg/d + CS

**Table 2:** Pathologic scoring criteria for liver tissue injury.

Pathological Changes	Score
Hepatocyte morphology is neat, with well-arranged hepatocyte cords and no inflammatory cell infiltration	0
Hepatocyte cords disorganized, occasional vacuoles, slight inflammatory cell infiltration	1
Hepatocytes have mild swelling, more vacuoles or inflammatory cell infiltration	2
Heavily swollen hepatocytes with large numbers of vacuoles or inflammatory and lymphocytic infiltrates	3
Large vacuolated lesions of hepatocytes with inflammatory necrosis and exudation of erythrocytes and lymphocytes	4

**Table 3:** Ingredients and nutrient composition of the sea cucumber viscera.

Ingredient	Content %
Crude protein	56.97 ± 0.73
Crude fat	24.27 ± 2.49
Ash	7.47 ± 0.15
Moisture	4.19 ± 0.95
Polysaccharide	4.76 ± 0.24
Calcium	0.27 ± 0.07
Phosphorus	0.78 ± 0.10

**Table 4:** Fatty Acid content of sea cucumber viscera.

Fatty Acid	Content (g·100 g^−1^)	Content %	Fatty Acid	Content (g·100 g^−1^)	Content %
C_11:0_	0.0039	0.03	C_18:3γ_	0.4085	3.42
C_12:0_	0.006	0.05	C_20:0_	0.2864	2.39
C_13:0_	0.1685	1.41	C_20:1_	0.0387	0.32
C_14:0_	0.0553	0.46	C_21:0_	0.093	0.78
C_14:1_	0.0217	0.18	C_20:3_	0.5596	4.68
C_15:0_	0.0182	0.15	C_22:0_	0.0286	0.24
C_15:1_	0.318	2.66	C_22:1_	2.1783	18.21
C_16:0_	2.3029	19.25	C_20:3_	0.281	2.35
C_16:1_	0.172	1.44	C_20:3_	0.2342	1.96
C_17:1_	1.2989	10.86	C_20:4_	0.7775	6.5
C_18:0_	0.1836	1.53	C_23:0_	0.7974	6.67
C_18:1_	0.3085	2.58	C_24:0_	0.3956	3.31
C_18:3 α_	0.3191	2.67	C_20:5_	0.7054	5.9
Saturated Fatty Acid	4.3393	36.28	Polyunsaturated Fatty Acid	3.3239	27.79
Unsaturated Fatty Acid	7.6212	63.72	ω-3	1.8020	15.07
Monounsaturated Fatty Acid	4.2973	35.93	ω-6	1.4203	11.87

## Data Availability

The raw data has been deposited in the National Center for Biotechnology Information database under the accession number PRJNA1311503 https://www.ncbi.nlm.nih.gov/bioproject/PRJNA1311503 (accessed on 27 August 2025).

## Supplementary Materials

The following supporting information can be downloaded at https://www.mdpi.com/article/10.3390/antiox14111355/s1: Figure S1: Effects of sea cucumber viscera on intestinal flora in mice. a. ASV/OTU Wayne plots. b. α-Diversity indices, i.e. Simpson, Shannon, and Pielou e; Table S1: Information on genes quantified by q-PCR and their primers; Table S2: Enteric microbiota in the top 15 genus-level abundance shares.
