# Supplementary material for: Unlocking the Antioxidant Potential of Sea Cucumber Viscera: Pre-Treatment Modulates the Keap1-Nrf2 Pathway and Gut Microbiota to Attenuate Cold Stress-Induced Oxidative Damage
Source: Antioxidants (Basel). 2025 Nov 13;14(11):1355. doi: 10.3390/antiox14111355 (PMC12649482; doi:10.3390/antiox14111355)
Supplement: Supplementary file 1 [file antioxidants-14-01355-s001.zip › antioxidants-3973600-supplementary.pdf]

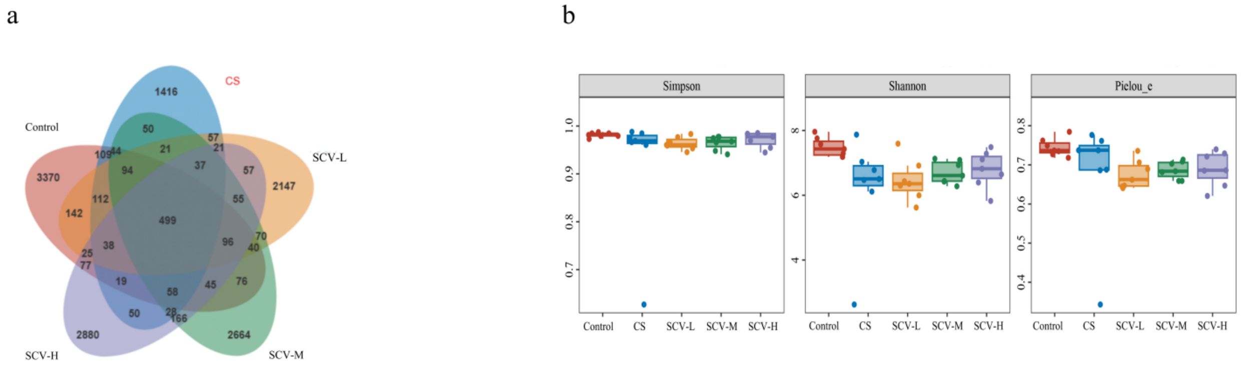

Supplementary Figure S1. Effects of sea cucumber viscera on intestinal flora in mice. a. ASV/OTU Wayne plots. b. α-Diversity indices, i.e. Simpson, Shannon, and Pielou_e.

Supplementary Table S1 Information on genes quantified by q-PCR and their primers

Genes	Primer sequences F:(5'-3')	Primer sequences R:(5'-3')	Amplicon (bp)
HSP70	CGCTCGACTCCTATGCCTTCA	GGCACTTGTCCACCACCTTC	102
HSP90	TGTTGCGGTACTACACATCTGC	GTCCTTGGTCTCACCTGTGATA	116
Keap1	TTGTTCTGCTGCCTCTTCTGC	GTGGGTGTTGCTGTCACCATG	199
Nrf2	AATATCCAGGGCAAGCGACTC	CAGCACATCCAGACAGACACC	153
HO-1	AGCGAAACAAGCAGAACCCA	ACCTCGTGGAGACGCTTTAC	166
GAPDH	GGTTGTCTCCTGCGACTTCA	TGGTCCAGGGTTTCTTACTCC	183

Supplementary Table S2 Enteric microbiota in the top 15 genus-level abundance shares

Item	Control	CS	SCV-L	SCV-M	SCV-H
Lactobacillus	0.08±0.07	0.23±0.26	0.22±0.10	0.21±0.08	0.08±0.07
Desulfovibrio	0.04±0.03	0.04±0.04	0.02±0.02	0.05±0.05	0.05±0.08
Allobaculum	0.00±0.00	0.00±0.00	0.08±0.06*	0.04±0.04	0.08±0.06*
Adlercreutzia	0.03±0.01	0.04±0.01	0.04±0.02	0.05±0.03	0.04±0.01
Corynebacterium	0.06±0.06	0.02±0.02	0.01±0.02	0.01±0.01	0.02±0.04
Prevotella	0.02±0.02	0.04±0.06	0.01±0.02	0.01±0.01	0.02±0.02
Oscillospira	0.04±0.02	0.02±0.02	0.01±0.01**	0.02±0.01	0.01±0.01*
[Prevotella]	0.01±0.00	0.02±0.03	0.02±0.01	0.01±0.01	0.03±0.03
Bacteroides	0.02±0.01	0.02±0.01	0.01±0.00	0.01±0.01	0.03±0.03
Ruminococcaceae_Ruminococc	0.02±0.01	0.01±0.01	0.01±0.01	0.01±0.01	0.02±0.02
Turicibacter	0.00±0.00	0.00±0.00	0.02±0.01*	0.03±0.02***	0.01±0.01
Coprococcus	0.01±0.01	0.01±0.02	0.01±0.01	0.02±0.02	0.01±0.01
Bifidobacterium	0.00±0.00	0.00±0.00	0.02±0.03	0.01±0.02	0.01±0.01
Akkermansia	0.00±0.00	0.00±0.00	0.03±0.04*	0.00±0.00	0.00±0.00
Jeotgalicoccus	0.01±0.00	0.01±0.01	0.00±0.00	0.00±0.00	0.02±0.03
Others	0.66±0.10	0.56±0.23	0.49±0.10	0.52±0.09	0.57±0.11

Note: Subgroups: control (Control), cold stress (CS), low-dose sea cucumber viscera group (SCV-L, 200 mg/kg/d), medium-dose sea cucumber viscera group (SCV-M, 400 mg/kg/d), and high-dose sea cucumber viscera group (SCV-H, 800 mg/kg/d); Results are presented as mean ± standard deviation (mean±SD,n=3).*P<0.05, **P<0.01 and ***P<0.001 vs Control.
